# Study on the application of percutaneous closed pleural brushing combined with cell block technique in the diagnosis of malignant pleural effusion

**DOI:** 10.1111/crj.13705

**Published:** 2023-09-29

**Authors:** Kang Wang, Xueting Hu, Yufang Chen, Xinglin Yi, Xianfeng Han, Duan Zhu, Bingjing Zhu, Hu Luo

**Affiliations:** ^1^ Department of Respiratory and Critical Care Medicine The First Affiliated Hospital of the Army Medical University Chongqing China; ^2^ Department of Geriatrics The First Affiliated Hospital of the Army Medical University Chongqing China

**Keywords:** carcinoembryonic antigen, cell block examination, closed pleural biopsy, closed pleural brushing, epidermal growth factor receptor, malignant pleural effusion

## Abstract

**Introduction:**

This study was to investigate the diagnostic value of percutaneous closed pleural brushing (CPBR) followed by cell block technique for malignant pleural effusion (MPE) and the predictive efficacy of pleural fluid carcinoembryonic antigen (CEA) for epidermal growth factor receptor (*EGFR*) mutations in lung adenocarcinoma patients with MPE.

**Methods:**

All patients underwent closed pleural biopsy (CPB) and CPBR followed by cell block examination. MPE‐positive diagnostic rates between the two methods were compared. Univariate and multivariate analyses were performed to determine factors influencing the *EGFR* mutations. Receiver operating characteristic (ROC) curve was used to analyze the predictive efficacy of pleural fluid CEA for *EGFR* mutations.

**Results:**

The cumulative positive diagnostic rates for MPE after single and twice CPBR followed by cell block examination were 80.5% and 89.0%, higher than CPB (45.7%, 54.3%) (*P* < 0.001). Univariate analysis showed that *EGFR* mutation was associated with pleural fluid and serum CEA (*P* < 0.05). Multivariate analysis showed that pleural fluid CEA was an independent risk factor for predicting *EGFR* mutation (*P* < 0.001). The area under the curve (AUC) of pleural fluid CEA for *EGFR* mutation prediction was 0.774, higher than serum CEA (*P* = 0.043), but no difference with the combined test (*P* > 0.05).

**Conclusion:**

Compared with CPB, CPBR followed by the cell block technique can significantly increase the positive diagnostic rate of suspected MPE. CEA testing of pleural fluid after CPBR has a high predictive efficacy for *EGFR* mutation in lung adenocarcinoma patients with MPE, implying pleural fluid extracted for cell block after CPBR may be an ideal specimen for genetic testing.

## Graphical abstract

Our findings confirm that closed pleural brushing followed by cell block technique has a high diagnostic rate for malignant pleural effusion. In addition, we also found that carcinoembryonic antigen testing of pleural fluid after closed pleural brushing has a high predictive efficacy for epidermal growth factor receptor mutation in lung adenocarcinoma patients with malignant pleural effusion.

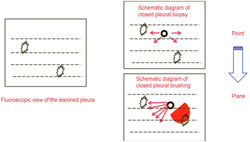

## 1 INTRODUCTION

Malignant pleural effusion (MPE) is caused by metastasis of malignant tumors from other sites to the pleura or malignant tumors originating in the pleura.^1^ The annual number of new MPE patients in the United States exceeds 150 000, with approximately 125 000 cases requiring hospitalization, and the annual treatment‐related costs exceed $5 billion, imposing a huge economic burden on patients and society.^2^, ^3^ MPE can be found in most tumors. Lung, breast, pleural mesothelioma, lymphoma, and ovarian cancer are all common causes of MPE, with lung cancer being the most common, accounting for about 1/3 of the cases.^4^ Malignant tumor patients with MPE have generally progressed to stage IV and have a poor prognosis. Depending on the type and stage of the tumor, the median survival time fluctuates from 3 to 12 months from the time of MPE diagnosis.^5^ Therefore, it is important to diagnose MPE early and take timely treatment.

Pathological malignancy changes observed in pleural biopsy tissue or finding malignant cells in the pleural fluid is the current gold standard for diagnosing MPE. Currently, pleural tissues are mainly obtained by percutaneous closed pleural biopsy (CPB), CT‐guided percutaneous pleural biopsy, and thoracoscopic pleural biopsy.^6^ Although CPB is simple and widely used, the diagnostic rate is only about 40%. The diagnostic rate of CT‐guided percutaneous pleural biopsy is about 87%, and that of thoracoscopic pleural biopsy is about 95%. However, these two methods are complicated to operate and costly and have a relatively high incidence of complications such as bleeding and pneumothorax, especially for elderly patients with more underlying diseases, so their promotion in clinical practice is limited.^7^, ^8^ Pleural fluid cytology (PFC) is mainly used to diagnose MPE by finding malignant tumor cells in pleural fluid, which has the advantages of being less invasive, economical, and rapid. However, interfered with by the small number of cells in the pleural fluid and uneven smear, the diagnosis rate of MPE by traditional cytology smear method is only 50%–60%.^9^ In recent years, the cell block technique has been further applied based on PFC, and then the disease is diagnosed by immunohistochemistry. This method has improved the diagnostic rate of MPE, but the lack of sufficient numbers of malignant cells greatly limits the value of the application of pleural fluid cell blocks.^10^

Among patients with MPE due to lung cancer, lung adenocarcinoma is the most common.^11^ Epidermal growth factor receptor tyrosine kinase inhibitors (*EGFR*‐TKIs) are the first‐line treatment option for patients with advanced lung adenocarcinoma carrying *EGFR* mutations.^12^ Therefore, genetic testing is important for those patients. Genetic testing is mainly performed by CT‐guided lung puncture to obtain specimens; in addition, blood and pleural fluid can also be used as test specimens.^13^ Carcinoembryonic antigen (CEA) is of great value in monitoring the recurrence and prognosis of lung adenocarcinoma.^14^ In patients with lung adenocarcinoma with pleural metastases, the predictive value of pleural CEA for *EGFR* mutations indirectly reflects the value of pleural fluid as a genetic test specimen. Lian et al.^13^ pointed out that the rate of detection of the *EGFR* gene was associated with tumor cell number. How can we use pleural fluid to obtain a relatively abundant number of tumor cells to provide a good cytological basis for the subsequent cell block technique and genetic testing?

Closed pleural brushing (CPBR) refers to the delivery of a cytobrush into the pleural cavity through a thoracentesis needle, which brushes the pleura over a larger area, a circular area with the length of the cytobrush (generally 2 cm) as the radius, providing easier access to diseased tissue on the pleura than CPB. In a few studies on CPBR at home and abroad, pleural brushing was performed after the pleural fluid was drained, and then smears were sent for cytological examination.^15^ However, this method not only easily damages the visceral pleura or lung but also fails to increase the number of cells in the pleural fluid. We envision that by brushing the cells from the diseased tissue into the pleural fluid through CPBR, the number of malignant cells in the pleural fluid can be greatly increased, providing a more adequate cytological basis for subsequent immunohistochemistry and genetic testing. Increase the positive rate of diagnosis and even the rate of genetic testing delivery. The purpose of this study was to investigate the diagnostic value of CPBR followed by cell block technique in MPE and the predictive value of pleural fluid CEA for *EGFR* mutations.

## 2 MATERIALS AND METHODS

### 2.1 Study subjects

Case data were collected consecutively from October 2018 to October 2020 from patients with MPE who visited the Southwest Hospital of the Third Military Medical University (Army Medical University). Inclusion criteria are as follows: (1) those aged between 18 and 85 years; (2) those with MPE diagnosed by histopathology; (3) those with pleural effusion localizable by ultrasound and ≥3 cm in thickness; (4) those with CPB followed by CPBR combined with cell block examination; and (5) those with confirmed lung adenocarcinoma followed by genetic testing. Exclusion criteria are as follows: (1) incomplete case data; (2) contraindicated for invasive operations such as thoracentesis and pleural biopsy; (3) with other serious cardiopulmonary diseases; and (4) mutations other than *EGFR* mutations, such as *ALK*, *HER2*, and *MET*. This study was approved by the Medical Ethics Committee of the First Affiliated Hospital of the Army Military Medical University (KY201992).

### 2.2 Closed pleural biopsy

The patients were informed of the precautions before the operation. Then, they were placed in a sitting position and marked after ultrasound localization. The puncture site was disinfected and toweled and then infiltrated with 2% lidocaine for anesthesia. The pleural biopsy Cope's needle (Shanghai Injection Needle Factory, China) was inserted into the pleural cavity, and two to three pieces of pleural tissue were taken out and fixed with 10% formaldehyde solution and sent for examination.

### 2.3 Closed pleural brushing

After the biopsy, a BF IT40 fiberoptic bronchoscopic cytobrush (Olympus, Japan) matching the inner diameter of the Cope's needle cannula was delivered into the pleural cavity through the cannula. During the brushing process, the direction of the Cope's needle cannula is constantly changed and the brush is slowly pumped back and forth to brush as wide an area as possible to “brush” more tumor cells into the pleural fluid. The operation should be gentle and slow, and the range of movement should not be too large. After the CPBR, 5 mL of pleural fluid was first extracted for pleural fluid CEA, and then 500 mL of pleural fluid was extracted for cytological examination.

### 2.4 Cell block

After 500‐mL pleural fluid was left to stand for 10–15 min, the lower half of 150‐mL liquid was removed and placed in a centrifuge tube, the centrifuge speed was adjusted to 2000 r/min and centrifuged for 5 min, and the supernatant was discarded. Fifteen milliliters of 10% formalin was added to the centrifuge tube, mixed well, and centrifuged (2000 r/min, 5 min), and the supernatant was discarded. An additional 30 mL of 95% ethanol was added and allowed to stand for 1–2 h. The precipitates were removed with forceps, dehydrated and paraffin‐embedded, and then made into cell blocks. The cell blocks were made into 3‐μm‐thick sections and then subjected to hematoxylin–eosin staining and immunohistochemistry. The results of the pathological examination were reported by the pathologist. If both methods (method1: CPB; method2: CPBR combined with cell block) were negative, a second puncture was performed 1 month later, and if both punctures were negative, other methods such as CT‐guided percutaneous pleural biopsy and thoracoscopic pleural biopsy were used for diagnosis.

### 2.5 Serum and pleural fluid CEA and genetic testing

Three milliliters of blood was drawn in the early morning fasting state, and 5 mL of pleural effusion was drawn after CPBR. Elecys2010 electrochemiluminescence assay was used for CEA detection according to the instructions. Tissue specimens or liquid specimens were used for gene detection by the next‐generation sequencing (NGS) method. NGS: Genomic DNA of the specimens was extracted using the QIAamp DNA Mini Kit (QIAGEN, Germany), and the operation was performed strictly according to the instructions. The IlluminaTruSeq kit (Illumina, USA) was used to construct DNA sequencing libraries, and specific probes were used to capture the target DNA fragments, and then the Illumina Hiseq 4000 sequencing platform (Illumina, USA) was used for sequencing with a read length of PE150 and sequencing depth of >500×. The raw data obtained were filtered and bioinformatically analyzed.

### 2.6 Statistical analysis

SPSS 23.0 (IBM Corp, Armonk, NY) statistical software was used for data analysis. The measurement data were first analyzed using the Kolmogorov–Smirnov test, and if the results showed conformity to a normal distribution, they were described using mean ± standard deviation (SD) and independent samples *t*‐test for comparison. If they did not conform to a normal distribution, the median and quartiles [M(P25, P75)] were used to describe them and the Mann–Whitney test for comparison. The count data were expressed as *n* (%) and the *χ* ^2^ test for comparison. The relationship between the factors and *EGFR* mutation status was analyzed using logistic regression models. Receiver operating characteristic (ROC) curves were used to compare the predictive efficacy of pleural fluid CEA and serum CEA for *EGFR* mutations. A *P*‐value of <0.05 was considered statistically significant.

## 3 RESULTS

### 3.1 General information of the included patients

A total of 164 patients with MPE were included: Eighty‐three males and 81 females; mean age (62.23 ± 12.61) years; 148 cases of lung adenocarcinoma, 12 cases of small cell lung cancer, two cases of squamous lung cancer, one case of ovarian cancer, and one case of malignant pleural mesothelioma (Table 1).

**TABLE 1:** General conditions of 164 patients with malignant pleural effusion.

General conditions	Mean ± SD, *n* (%)
Gender	
Male	83 (50.6%)
Female	81 (49.4%)
Age	
Mean (years)	62.23 ± 12.61
Range (years)	27 ~ 85
≥60 years old	102 (62.2%)
<60 years old	62 (37.8%)
Tumor types	
Lung adenocarcinoma	148 (90.2%)
Small cell lung cancer	12 (7.3%)
Lung squamous carcinoma	2 (1.2%)
Ovarian Cancer	1 (0.6%)
Malignant pleural mesothelioma	1 (0.6%)

Abbreviation: SD, standard deviation.

### 3.2 Comparison of positive diagnostic rates between CPB and CPBR followed by cell block

The positive diagnostic rates of single CPB and CPBR followed by cell block were 45.7% and 80.5%, respectively (*P* < 0.001). The cumulative positive diagnostic rate of two times CPBR followed by cell block was 89.0%, which was significantly higher than that of two times CPB (54.3%) (*P* < 0.001). The positive rate of performing two times of examinations was higher than that of a single examination, and increasing the number of delivery examinations could improve the positive rate (Tables 2 and 3).

**TABLE 2:** Number of CPB and positive diagnostic rate in 164 patients with malignant pleural effusion (*n*, %).

Number of CPB	Positive cases	Cumulative positive cases	Cumulative positivity rate (%)
First time	75	75	45.7
Second time	14	89	54.3

Abbreviation: CPB, closed pleural biopsy.

**TABLE 3:** Number of CPBR followed by cell block and positive diagnostic rate in 164 patients with malignant pleural effusion (*n*, %).

Number of CPBR followed by cell block	Positive cases	Cumulative positive cases	Cumulative positivity rate (%)
First time	132	132	80.5
Second time	14	146	89.0

Abbreviation: CPBR, closed pleural brushing.

### 3.3 Univariate analysis of *EGFR* mutation status in patients with lung adenocarcinoma with MPE

Of the 164 patients with MPE, 148 were lung adenocarcinoma, and all patients with lung adenocarcinoma underwent genetic testing. Among them, 49 patients had an *EGFR* mutation and 99 patients had no *EGFR* mutation, and the mutation rate was 33.1%. They were divided into *EGFR* mutated group and *EGFR* unmutated group according to the mutation status. Univariate analysis showed that *EGFR* mutation was associated with pleural fluid CEA and serum CEA (*P* < 0.05), but not with gender, age, smoking history, bone metastasis, or brain metastasis (*P* > 0.05) (Table 4).

**TABLE 4:** Univariate analysis of *EGFR* mutation status in patients with lung adenocarcinoma with MPE [Mean ± SD, *n* (%)].

Clinical and pathological features	*EGFR* mutated group (*n* = 49)	*EGFR* unmutated group (*n* = 99)	*χ* ^2^/*t*	*P*
Gender				
Male	20 (40.8%)	50 (50.5%)	1.234	0.267
Female	29 (59.2%)	49 (49.5%)		
Age				
≥60 years old	33 (67.3%)	56 (56.6%)	1.589	0.207
<60 years old	16 (32.7%)	43 (43.4%)		
Smoking history				
Yes	16 (32.7%)	45 (45.5%)	2.217	0.136
No	33 (67.3%)	54 (54.5%)		
Bone metastasis				
Yes	24 (49.0%)	33 (33.3%)	3.388	0.066
No	25 (51.0%)	66 (66.7%)		
Cranial metastases				
Yes	11 (22.4%)	13 (13.1%)	2.094	0.148
No	38 (77.6%)	86 (86.9%)		
Pleural fluid CEA (ng/mL)	84.07 ± 22.93	18.18 ± 2.59	2.855	0.006
Serum CEA (ng/mL)	55.35 ± 10.96	28.87 ± 4.90	2.205	0.031

Abbreviations: CEA, carcinoembryonic antigen; *EGFR*, epidermal growth factor receptor; MPE, malignant pleural effusion.

### 3.4 Multivariate analysis of *EGFR* mutation status in patients with lung adenocarcinoma with MPE

In univariate analysis, *EGFR* mutation was associated with pleural fluid CEA and serum CEA, and several large clinical trials concluded that gender and smoking history were also associated with *EGFR* mutation, so we included gender, smoking history, pleural fluid CEA, and serum CEA in multivariate logistic regression analysis. The results showed that pleural fluid CEA was an independent predictor of *EGFR* mutation in patients with lung adenocarcinoma with MPE (*OR*: 1.024, 95% CI: 1.011–1.037, *P* < 0.001) (Table 5).

**TABLE 5:** Multivariate analysis of *EGFR* mutation status in patients with lung adenocarcinoma with MPE.

Factors	*β*	*SE*	Wald *x* ^2^	*OR*	95% CI	*P*
Gender	−0.505	0.741	0.464	0.604	0.141–2.578	0.496
Smoking history	0.903	0.767	1.384	2.467	0.548–11.101	0.239
Serum CEA	0.003	0.002	3.704	1.003	1.000–1.007	0.054
Pleural fluid CEA	0.024	0.006	13.974	1.024	1.011–1.037	<0.001

Abbreviations: CEA, carcinoembryonic antigen; CI, confidence interval; *EGFR*, epidermal growth factor receptor; MPE, malignant pleural effusion; OR, odd ratio; SE, standard error.

### 3.5 Analysis of the predictive efficacy of pleural fluid CEA, serum CEA, and pleural fluid CEA/serum CEA combined with pleural fluid CEA for *EGFR* mutations in lung adenocarcinoma patients with MPE

**FIGURE 1:**
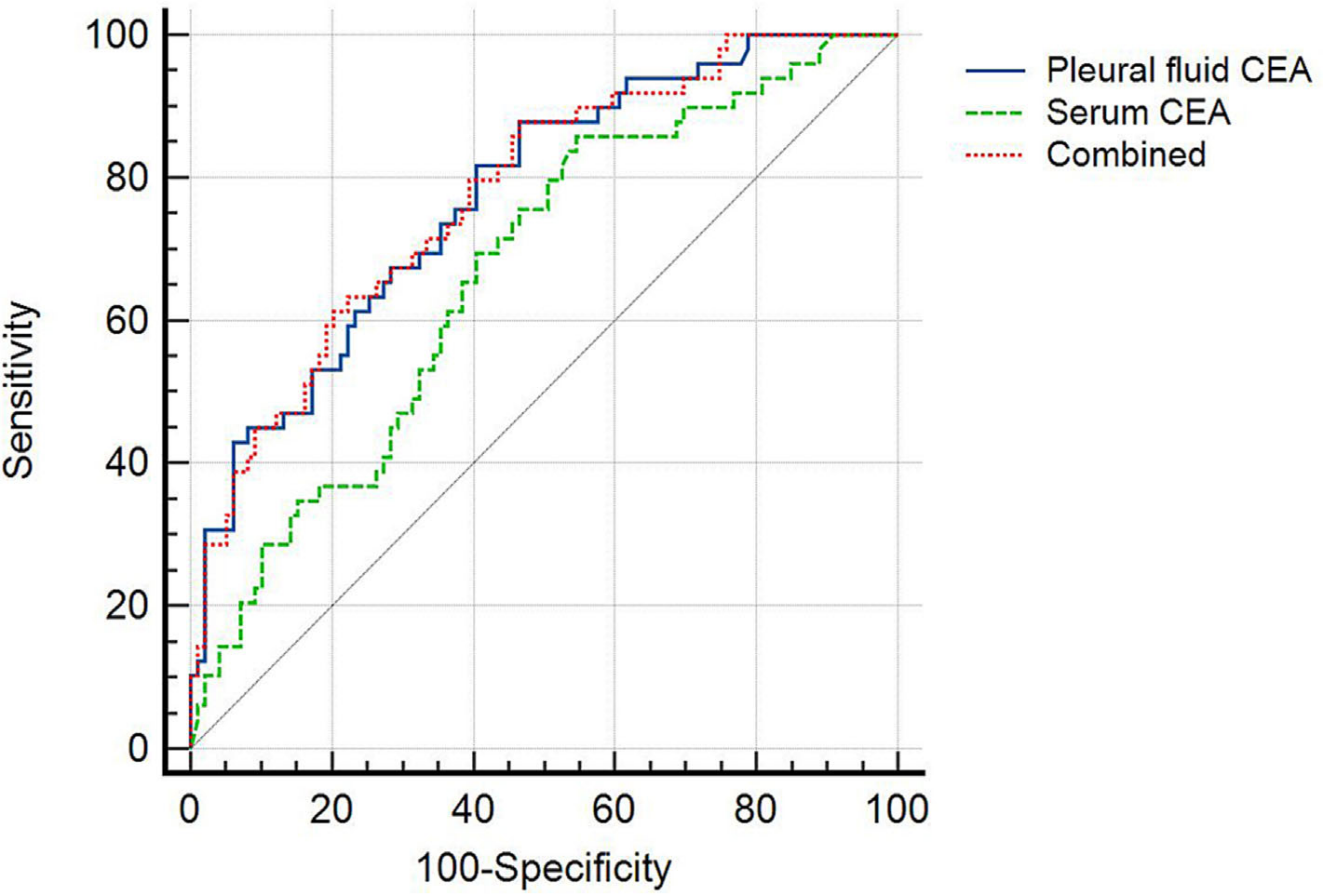
ROC comparison of pleural fluid CEA, serum CEA, and pleural fluid CEA/serum CEA combined with pleural fluid CEA for the prediction of *EGFR* mutations in lung adenocarcinoma patients with malignant pleural effusion (CEA, carcinoembryonic antigen).

The area under the curve (AUC) of pleural fluid CEA for the prediction of *EGFR* mutation in lung adenocarcinoma patients with MPE was 0.774, with a cut‐off value of 7.94 ng/mL, sensitivity of 87.76%, and specificity of 53.54% (*P* < 0.001). The AUC of serum CEA for *EGFR* mutation prediction was 0.668 and the cut‐off value was 5.10 ng/mL, with a sensitivity of 85.71% and specificity of 45.45% (*P* < 0.001). The AUC value of pleural fluid CEA was greater than that of serum CEA (*Z* = 2.027, *P* = 0.043). Based on pleural fluid CEA, we combined the ratio of pleural fluid CEA to serum CEA to jointly predict *EGFR* mutations and finally obtained an AUC value of 0.777. There was no difference compared with pleural fluid CEA alone (*Z* = 0.475, *P* = 0.635) (Table 6 and Figure 1).

**TABLE 6:** Comparison of the predictive efficacy of pleural fluid CEA, serum CEA and pleural fluid CEA/serum CEA combined with pleural fluid CEA for *EGFR* mutations in patients with lung adenocarcinoma with MPE.

	AUC	95% CI	Cut‐off value	Sensitivity (%)	Specificity (%)	*P*
Pleural fluid CEA	0.774	0.696–0.852	7.94	87.76	53.54	<0.001
Serum CEA	0.668	0.578–0.758	5.10	85.71	45.45	<0.001
Pleural fluid CEA/serum CEA combined with pleural fluid CEA	0.777	0.701–0.841	‐	87.79	53.56	<0.001

Abbreviations: AUC, area under the curve; CEA, carcinoembryonic antigen; CI, confidence interval; *EGFR*, epidermal growth factor receptor; MPE, malignant pleural effusion.

### 3.6 Pleural fluid CEA and *EGFR* mutation type

**FIGURE 2:**
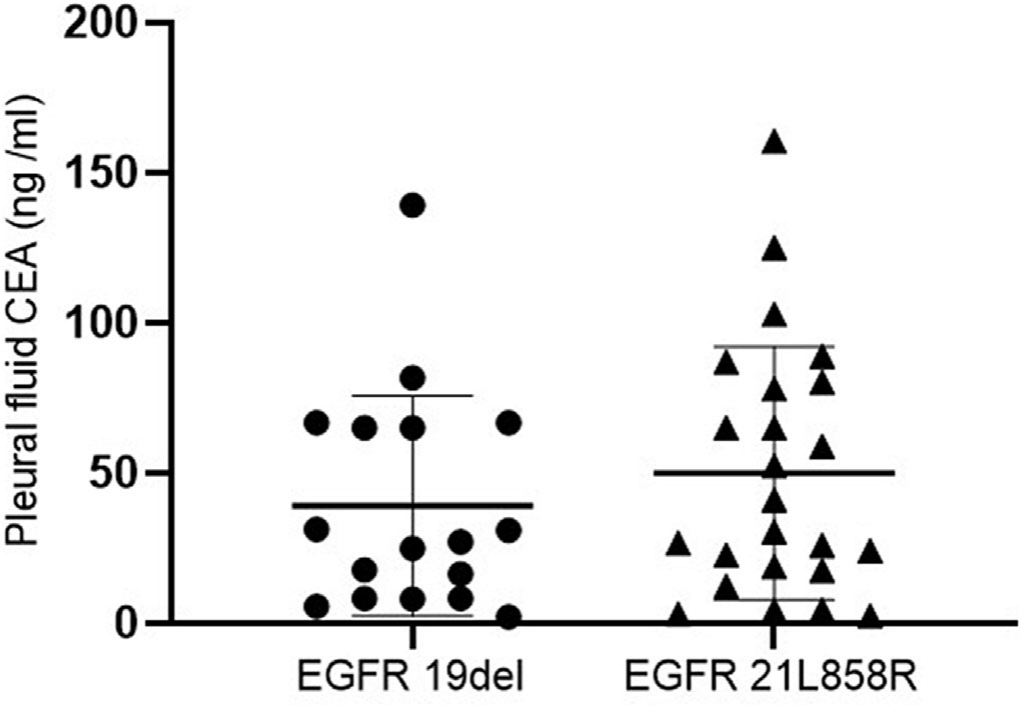
Comparison of pleural fluid CEA in patients with *EGFR* 19del and *EGFR* 21L858R mutation (*EGFR*, epidermal growth factor receptor; CEA, carcinoembryonic antigen).

Among 148 patients with lung adenocarcinoma, 49 cases had *EGFR* mutations. The common mutations were 41 cases, including 17 cases of *EGFR* 19del mutation and 24 cases of *EGFR* 21L858R mutation. Rare mutations were found in eight cases (three cases of *EGFR* 20 insertion; two cases of *EGFR* 21L861Q; one case of *EGFR* 18G719X, *EGFR* 18L718Q, and *EGFR* 18G719C). The mean values of pleural fluid CEA of patients with *EGFR* 19del mutation and *EGFR* 21L858R mutation were (39.11 ± 36.69) ng/mL and (49.95 ± 42.15) ng/mL, respectively, with no difference (*t* = 0.855, *P* = 0.398) (Figure 2).

## 4 DISCUSSION

MPE is the second cause of death among all exudative pleural effusions.^16^ Easy and accurate diagnosis of MPE not only can quickly clarify the cause and take corresponding treatment measures but also reduce the risk associated with patients who fail to make a clear diagnosis and turn to CT‐guided percutaneous lung biopsy, thoracoscopic direct view pleural biopsy, and other clinical invasive operations to obtain pathological specimens and reduce the corresponding medical costs. In this study, the positive diagnostic rate of MPE was compared between CPB and CPBR followed by cell block, and it was found that CPBR followed by cell block greatly improved the positive diagnostic rate of MPE. In addition, after CPBR, we extracted pleural fluid for CEA testing to investigate the predictive efficacy of pleural fluid CEA for *EGFR* mutations and found that pleural fluid CEA has a high predictive value for *EGFR* mutations, indirectly reflecting the importance of pleural fluid for genetic testing. Pleural fluid extracted for cell block after CPBR is expected to be an ideal specimen for genetic testing.

Malignant tumor cells are unevenly distributed after metastasis to the pleura. CPB can only get a few points of pleural tissue, elderly patients are not easily hooked due to the brittle pleura, and those who are not experienced enough in operation often get muscle tissue, which leads to a low positive rate of pleural biopsy. A retrospective study by Zhang et al.^17^ included a total of 644 patients with exudative pleural effusion, of which 509 were MPE, and the positive diagnostic rate of CPB for the diagnosis of MPE was 51.5%. The positive diagnostic rate of CPB for the diagnosis of MPE in our study was 45.7%, similar to the results of the above study. The positive diagnostic rates of CPB for MPE in the studies of Pereyra et al.^18^ and Jakubec et al.^19^ were 59.2% and 63.1%, respectively. Case selection bias, differences in the level of CPB operation, and differences in ethnicity can cause variability in the positive diagnostic rate. PFC is widely used in clinical practice, and a study by Basso et al.^20^ noted that the positive diagnostic rate of MPE by PFC was 55.0%. The use of the cell block technique on top of PFC can improve the positive rate of MPE diagnosis, and Miyoshi et al.^21^ concluded that the positive diagnosis rate of MPE using the cell block technique on top of PFC reached 71.4%. The diagnostic efficacy of the cytology‐based cell block technique depends on the number of tumor cells obtained in the pleural fluid. In patients with suspected MPE, our conventional approach has been to perform a CPB followed by a pleural fluid cell block. However, in this situation, the number of tumor cells in the pleural fluid is low, which limits the diagnostic efficacy of the cell block technique. During pleural brushing, the range of motion of the cell brush in the pleural cavity is significantly expanded compared with biopsy, especially for those areas that are difficult to reach by biopsy and can be brushed in a fan‐shaped surface, making it easier to obtain lesion specimens than pleural biopsy. Aksoy et al.^22^ compared the diagnostic efficacy of CPBR, CPB, and PFC for MPE and showed that CPBR had a positive diagnostic rate of 57% for MPE, which was higher than CPB and PFC. However, domestic and international studies on CPBR have performed pleural brushing after pleural fluid is drained, followed by smears for cytological examination. This method does not essentially involve the cell block technique, and pleural fluid drained can cause reopening of the lungs, and there is a high risk of injury to the visceral pleura or even the lungs when performing brushing in this situation. Therefore, the high operative risk may also explain the paucity of clinical studies on CPBR. To address these issues, we believe that pleural brushing may increase the number of tumor cells in pleural fluid, and then extraction of pleural fluid for cell block may help improve the diagnostic rate of MPE. In our study, we first performed CPB, then CPBR, and finally extracted pleural fluid for cell block. Of the 164 patients with MPE, CPBR followed by cell block confirmed the diagnosis in 132 cases at the first examination, with a positive diagnostic rate of 80.5%, significantly higher than that of 45.7% for CPB (*P* < 0.001). Patients who were negative for both methods after the first examination were examined a second time, and the results showed that the cumulative positive rate of twice examinations was higher than that of the single examination, suggesting that increasing the number of tests could improve the positive rate.

Of the 164 patients with MPE in this study, 148 had lung adenocarcinoma, accounting for 90.2% of the total, suggesting that patients with lung adenocarcinoma may be more likely to develop pleural metastases. Patients with lung adenocarcinoma have a high rate of positive *EGFR* mutations, especially in Asian, nonsmoking, and female populations, where the mutation rate of *EGFR* is as high as 40%–50%.^23^ For genetic testing of patients with advanced lung adenocarcinoma, CT‐guided lung biopsy is mainly used to obtain specimens, and the utilization rate of pleural fluid is relatively low. However, this method of obtaining specimens for genetic testing is not only invasive but also expensive. To investigate the value of pleural fluid after pleural brushing for genetic testing and whether the cell block specimen is expected to be an ideal specimen for genetic testing, we measured the level of CEA in pleural fluid after pleural brushing and analyzed the predictive efficacy of pleural fluid CEA for *EGFR* mutations to indirectly reflect the value of pleural fluid. Our study showed that *EGFR* mutation was associated with pleural fluid CEA and serum CEA in univariate analysis, but not with gender, age, smoking history, bone metastasis, or cranial metastasis. Pleural fluid CEA was an independent risk factor for predicting *EGFR* mutation in lung adenocarcinoma patients with MPE in multivariate logistic regression analysis (*OR*: 1. 024, 95% CI: 1. 011–1. 037, *P* < 0.001), indicating that pleural fluid CEA is of significant value for predicting *EGFR* mutation. The results did not highlight the advantage of high *EGFR* mutation rates in the nonsmoking, female population, possibly due to bias from the small number of cases we included. Hernandez et al.^24^ found that pleural fluid CEA had a higher sensitivity and specificity of 49% and 98% for the diagnosis of MPE than serum CEA (33% and 97%). We used ROC curve analysis to compare the efficacy of pleural fluid CEA with serum CEA for *EGFR* mutation prediction, and the AUC of pleural fluid CEA for *EGFR* mutation prediction was 0.774, with a sensitivity of 87.76% and specificity of 53.54%, higher than that of serum CEA (*Z* = 2.027, *P* = 0.043). Our findings are consistent with the above study; this indicates that pleural fluid CEA has a higher value in predicting *EGFR* mutations compared with serum CEA, which further highlights the value of pleural fluid for genetic testing. To verify whether the ratio of pleural fluid CEA to serum CEA can increase the predictive value of pleural fluid CEA for *EGFR* mutation, we combined pleural fluid CEA/blood CEA based on pleural fluid CEA to predict *EGFR* mutation. The results showed that the predictive value of the combination was not superior to that of pleural fluid CEA alone, indicating that pleural fluid CEA alone could achieve the desired predictive effect for *EGFR* mutations. We further analyzed the pleural fluid CEA levels between different mutation types of common *EGFR* mutations and found that patients with *EGFR* 21L858R mutations had slightly higher pleural fluid CEA levels than those with *EGFR* 19del mutations, but the difference was not statistically significant (*P* > 0.05). We will expand the sample size for validation in the next step. The present study also has some limitations. We did not have a sufficient number of cases, and we did not directly compare the efficacy of cell block specimens made after pleural brushing with that of CT‐guided lung biopsy specimens for the detection of *EGFR* mutations. In the next step, we will expand the sample size and conduct a comparative study of cell block specimens made after pleural brushing and CT‐guided lung biopsy specimens.

## 5 CONCLUSIONS

Compared with CPB, CPBR followed by cell block technique can significantly increase the positive diagnostic rate of suspected MPE and reduce the risk associated with patients who are referred to CT‐guided percutaneous lung biopsy, fiberoptic bronchoscopic mucosal biopsy, and other clinically invasive operations for failure to make a definitive diagnosis. The test of pleural fluid CEA after CPBR has high predictive efficacy for *EGFR* mutation in lung adenocarcinoma patients with MPE, which indirectly reflects the importance of pleural fluid for genetic testing, and pleural fluid extracted for cell block after CPBR is expected to be an ideal sample for genetic testing.

## AUTHOR CONTRIBUTIONS

*Conception and design*: Kang Wang and Hu Luo. *Methodology*: Xueting Hu, Duan Zhu, and Bingjing Zhu. *Data Collection*: Xianfeng Han, Xinglin Yi, and Yufang Chen. *Data analysis and graphing*: Kang Wang, Xueting Hu, and Yufang Chen. *Manuscript Writing*: Kang Wang, Xueting Hu, and Hu Luo. *Manuscript revision and editing*: Hu Luo, Duan Zhu, and Bingjing Zhu. *Research supervision*: Hu Luo. All authors contributed to the article and approved the submitted version.

## CONFLICT OF INTEREST STATEMENT

The authors declare that the research was conducted in the absence of any commercial or financial relationships that could be construed as a potential conflict of interest.

## ETHICS STATEMENT

This study was approved by the Medical Ethics Committee of the Southwest Hospital of the Third Military Medical University (Army Medical University) (approval number: KY201992). The requirement for written informed consent was waived due to the retrospective study design.

## Data Availability

The raw data supporting the conclusions of this article will be made available by the corresponding author, without undue reservation.
